# How exotic plants integrate into pollination networks

**DOI:** 10.1111/1365-2745.12310

**Published:** 2014-09-18

**Authors:** Daniel B Stouffer, Alyssa R Cirtwill, Jordi Bascompte, Ignasi Bartomeus

**Affiliations:** 1 School of Biological Sciences, University of Canterbury, Private Bag 4800Christchurch, 8140, New Zealand; 2 Integrative Ecology Group, Estación Biológica de Doñana (EBD-CSIC)c/ Américo Vespucio s/n, 41092, Sevilla, Spain

**Keywords:** coevolution, competition, extinction, generalists, indirect facilitation, invasion ecology, mutualistic networks, nestedness, plant–animal interactions, specialization

## Abstract

**Summary:**

1. There is increasing world-wide concern about the impact of the introduction of exotic species on ecological communities. Since many exotic plants depend on native pollinators to successfully establish, it is of paramount importance that we understand precisely how exotic species integrate into existing plant–pollinator communities.
2. In this manuscript, we have studied a global data base of empirical pollination networks to determine whether community, network, species or interaction characteristics can help identify invaded communities.
3. We found that a limited number of community and network properties showed significant differences across the empirical data sets – namely networks with exotic plants present are characterized by greater total, plant and pollinator richness, as well as higher values of relative nestedness.
4. We also observed significant differences in terms of the pollinators that interact with the exotic plants. In particular, we found that specialist pollinators that are also weak contributors to community nestedness are far more likely to interact with exotic plants than would be expected by chance alone.
5. *Synthesis*. By virtue of their interactions, it appears that exotic plants may provide a key service to a community's specialist pollinators as well as fill otherwise vacant ‘coevolutionary niches’.

## Introduction

There is increasing world-wide concern about the impact of introductions of exotic species on ecological communities (Rejmánek & Richardson 1996; Sax & Gaines 2008). In particular, the successful introduction of exotic species can create effects that cascade through existing communities, for example by creating novel communities that are more resistant to restoration efforts (Aizen, Morales & Morales 2008; Tylianakis 2008; Valdovinos *et al*. 2009; Traveset *et al*. 2013). Given the breadth of their impacts (Vilà *et al*. 2011), it is of paramount importance that we develop stronger theories with which to better predict the risk of future invasions (Rejmánek & Richardson 1996).

A key first barrier that any exotic species must overcome prior to establishment is competition with endemic species for existing resources (Levine *et al*. 2003; Ricciardi & Atkinson 2004; Powell, Chase & Knight 2011). Indeed, since many community properties, such as total species richness, are considered to be reasonable proxies for the amount of competition in a community (Levine 2000; Naeem *et al*. 2000; Strauss, Webb & Salamin 2006; Hayes & Barry 2008; Cadotte, Hamilton & Murray 2009), it is widely expected that they provide key indicators of invasibility. Many exotic species also depend on native pollinators to successfully establish (Memmott & Waser 2002; Aizen, Morales & Morales 2008; Bartomeus, Vilà & Santamaría 2008), and pollinators may be a key resource over which plants compete (Levin & Anderson 1970; Mosquin 1971; Moeller 2004; Bastolla *et al*. 2009; Jakobsson, Padrón & Traveset 2009; Mitchell *et al*. 2009).

More generally, the mutualistic interactions that occur between plants and pollinators are known to play a critical role in overall biodiversity maintenance (Bond 1994; Bascompte & Jordano 2007; Bastolla *et al*. 2009). As a result, it has also been suggested that the overarching structure of plant–pollinator mutualistic networks might also play a role in determining the invasibility of a community (Bartomeus, Vilà & Santamaría 2008; Padrón *et al*. 2009; Traveset *et al*. 2013). There are multiple hypotheses for how the structure of mutualistic networks can facilitate species coexistence (Bascompte, Jordano & Olesen 2006; Bascompte & Jordano 2007; Bascompte 2009). At the whole community level, it appears that both network connectance – the number of possible interactions that are actually observed in a community – and nestedness – a measure of whether these interactions are organized such that specialists interact with proper subsets of the species with whom generalists interact – are strongly related to community stability and persistence (Bastolla *et al*. 2009; Thébault & Fontaine 2010; Allesina & Tang 2012; Saavedra & Stouffer 2013). Echoing these results at the level of individual species, a species' degree – its total number of mutualistic interactions – and its nestedness contribution – whether or not those interactions increase or decrease community nestedness – are strongly related to that species' vulnerability to extinction (Thébault & Fontaine 2010; Saavedra *et al*. 2011; Allesina 2012; James, Pitchford & Plank 2012).

To complicate matters further, there is increasing evidence that local drivers of endemic biodiversity can also lead to increased exotic diversity (Elton 1958; Dukes & Mooney 1999; Kennedy *et al*. 2002; Levine *et al*. 2003). In California, for example, more diverse native plant communities also contained more non-native species (Kruger *et al*. 1989; Levine & D'Antonio 1999). This suggests that studies which take an entirely local focus may overlook less system-specific mechanisms that lead to the successful integration of exotic plants into pollination networks (Memmott & Waser 2002; Moragues & Traveset 2005; Bartomeus, Vilà & Santamaría 2008; Jakobsson, Padrón & Traveset 2009). Consequently, we attempt to take a more ‘macroecological’ perspective in this study and explore a global data set of empirical pollination networks – some of which include exotic plants and some that do not. In adopting this perspective, we have opted to focus on whether a suite of community, network, species or interaction properties show characteristic patterns while sidestepping the question of their root causes and/or consequences. Nevertheless, our primary objective was to develop further insight into two key questions at the core of invasion ecology (Rejmánek & Richardson 1996): What makes some communities more likely to be invaded, and what allows some species to establish more successfully than others?

## Materials and methods

### Empirical pollination networks

**Figure 1:**
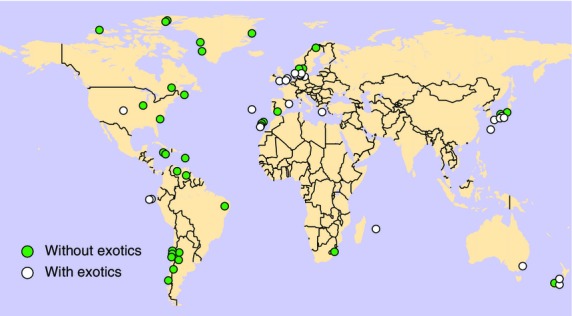
Map of the empirical pollination networks studied here. Green circles indicate the location of pollination networks that did not have exotic plants present while white circles indicate pollination networks with exotic species present.

Here we analyse a data set comprised of 59 plant–pollinator mutualistic networks from a wide range of locations around the globe and with diverse species assemblages (Web of Life, available at http://www.web-of-life.es; Fig.1 and Table S1 in Supporting Information). The interaction structures of each of the empirical networks are based on observed patterns of visitation of flowering plants by their insect pollinators. Of the 59 networks, 39 are qualitative networks where each interaction solely indicates presence or absence and 20 are quantitative networks in which each interaction has a ‘weight’ that corresponds to the observed frequency of visits (Jordano 1987).

### Identification of exotic species

In order to identify the exotic plant species across each of the empirical networks, we cross-referenced each species' name and network location (i.e. country) with its classification in both the Global Invasive Species Database (GISD; http://www.issg.org/database/welcome/) and the Global Invasive Species Information Network database (GISIN; http://www.gisin.org). In the GISD, species are classified as ‘Native’ or ‘Alien’; in the GISIN, the equivalent classifications are ‘Indigenous’ or ‘Exotic’. We considered all species as exotic if they were so classified in either (or both) of the two data bases. Lastly, note that records in GISIN classify species as different degrees of ‘Harmful’ as an indication of the species' impact in the community: ‘Yes’, ‘Potentially’ and ‘No’. Under this classification, invasive species should return ‘Harmful = Yes’; therefore, to capture as many species as possible within our data set, we consider here all non-native species regardless of their ‘Harmful’ classification. It is also for this reason that we refer to these species as exotic as opposed to invasive, recognizing that the definition of what precisely constitutes invasive is open to interpretation (Colautti & MacIsaac 2004).

Unfortunately, not all plants in the data set were identified at the species level. To err on the side of caution, these plants were never considered as exotic since it was technically impossible to determine their exact status. Furthermore, not all known exotic or invasive species can be found in these data bases (e.g. one of the focal species of Bartomeus, Vilà & Santamaría 2008). Nevertheless, matching all plants across the same data bases provides the most accurate and *reproducible* methodology that also controls for potential bias in the final results.

### Comparison of community-level properties

In order to determine whether communities with exotic plants differed in any characteristic manner, we calculated five properties related to community composition: plant richness *P*, pollinator richness *A*, total species richness *S* = *A* + *P*, the ratio *R* = *A*/*P* of pollinator to plant richness and the phylogenetic diversity of the plant community (as quantified by the average distance from all plants to their most recent common ancestor). We decided to focus on these five properties because each provides a proximate measure of community saturation and/or niche (or resource) availability, both of which have been argued to influence the ability of exotic species to establish in a novel community (Levine 2000; Strauss, Webb & Salamin 2006; Hayes & Barry 2008; Cadotte, Hamilton & Murray 2009). All community-level comparisons were made using a Kruskal–Wallis rank sum test.

Given an observed plant–pollinator interaction network, the first four properties are straightforward to determine. To calculate the last property, we first constructed each community's phylogenetic tree using the phylomatic ‘mega-tree’ (version R20120829) which defines relationships between higher plants (Webb, Ackerly & Kembel 2008). We then dated nodes across this tree according to Wikström, Savolainen & Chase (2001) and used the branch length adjustment algorithm BLADJ (Webb, Ackerly & Kembel 2008) to estimate the age of all remaining, undated nodes. Though this procedure implies that ages within our phylogenies should be treated as approximations (Beaulieu *et al*. 2007), previous analysis indicates marked improvements of phylogenetic analyses when even a limited number of nodes are properly dated (Webb 2000).

### Comparison of network-level properties

In order to determine whether networks with exotic plants differed in any characteristic manner, we calculated five properties related to the plant–pollinator network structure: overall network connectance *C* = *L*/(*PA*), average number of interactions per plant *C*_*P*_ = *L*/*P*, average number of interactions per pollinator *C*_*A*_ = *L*/*A*, nestedness *N* and relative nestedness N*. Here, *L* is the total number of mutualistic interactions observed in the community. We measured nestedness *N* using the metric NODF since it accounts for potential bias introduced by network size and topology compared with alternative measures (Almeida-Neto *et al*. 2008), and we measured relative nestedness – the degree to which nestedness compares to the amount expected at random – following Bascompte *et al*. (2003). Though network attributes, such as connectance, may not always be perfect indicators of community status (Heleno, Devoto & Pocock 2012), each of the five properties selected here has previously been shown to be associated with increased community stability (Allesina & Tang 2012), persistence (Saavedra *et al*. 2011; Saavedra & Stouffer 2013) and a community's overall ability to support higher levels of biodiversity (Bastolla *et al*. 2009). All network-level comparisons were made using a Kruskal–Wallis rank sum test.

### Comparison of species-level properties

In order to determine whether the exotic plants themselves differed in any characteristic manner, we calculated three properties with which to compare them to other plant species within the same network. For each plant species *i*, this included its rank *d*_*i*_ of phylogenetic ‘uniqueness’, the rank *k*_*i*_ of its degree and the rank *c*_*i*_ of its nestedness contribution (Saavedra *et al*. 2011). Here, we define the phylogenetic uniqueness of species *i* as the average phylogenetic distance between it and all other plants in its community. We quantified each species' nestedness contribution as the degree to which the observed nestedness of the network changes when randomizing just the interactions of the focal species (Saavedra *et al*. 2011). As with our analysis of relative nestedness, we randomized individual species' interactions according to Bascompte *et al*. (2003). We chose to study phylogenetic relatedness because it underpins key theories regarding community saturation and invasibility (Strauss, Webb & Salamin 2006; Fridley 2010). In addition, multiple theoretical studies indicate that having many connections and/or having a low nestedness contribution can reduce a species' vulnerability to extinction (Saavedra *et al*. 2011; Allesina 2012; Saavedra & Stouffer 2013).

All ranks were normalized within each network to vary between 0 and 1 such that a value of 0 corresponded to the smallest value in the network (e.g. the most phylogenetically typical) and a value of 1 corresponded to the largest value in the network (e.g. the most phylogenetically distinct). Because of the relationship between species abundance and network properties (Vázquez *et al*. 2007), species with low-rank degree can also be thought of as rare species. We chose to analyse ranks of all of these properties because the distributions of the raw values of each can show considerable variation across empirical networks (Jordano, Bascompte & Olesen 2003; Rezende *et al*. 2007; Saavedra *et al*. 2011). All species-level comparisons were made using a Kruskal–Wallis rank sum test.

### Comparison of interaction-level properties

To determine whether pollinators' interactions with exotic plants showed any characteristic differences from those with native plants, we calculated three interaction-level properties. For every observed interaction between a plant *i* and a pollinator *j*, we calculated the dependence 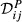 of the plant on the pollinator (i.e., the proportion of pollinator visits to *i* that come from pollinator *j*) and the corresponding dependence 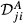 of the pollinator on the plant (Vázquez, Morris & Jordano 2005; Bascompte, Jordano & Olesen 2006). It has previously been argued that some variation in species' dependences can arise because of inherent differences in species abundance (Vázquez *et al*. 2007). Therefore, we also calculated each interactions' ‘preference’ Γ_*ij*_ which quantifies the degree to which the interaction deviates from a random-encounter (or mass-action) model (Staniczenko, Kopp & Allesina 2013). Here, a value of Γ_*ij*_ > 1 indicates that the interaction is preferred – in that it occurs more often than expected at random – whereas Γ_*ij*_ < 1 indicates that the interaction is less preferred.

Note that the calculation of dependences and interaction preferences requires prior information about relative interaction weights. Therefore, all interaction-level comparisons were restricted to the quantitative empirical networks in our data set. To statistically compare species' dependences, we used a binomial regression (Quinn & Keough 2002) with proportional visitation as the dependent variable and the plant species' classification (as native or exotic) as the independent variable; this regression was performed separately for plants and for pollinators. We compared interaction preferences using a simple linear regression with log-preferences as the independent variable and the plant species' classification as the independent variable.

### Comparison of pollinator properties

We lastly explored whether there were characteristic differences in terms of the pollinators that interacted with exotic plants. In particular, we quantified (i) whether more generalist pollinators showed a tendency to interact with exotic plants and (ii) whether pollinators that are stronger contributors to nestedness tended to interact with the exotic plants. In addition, we wanted to assess whether any relationship in the empirical data is not only statistically significant but also significantly different from what we would expect at random. The appositeness of this distinction can most easily be understood when considering the case of specialist vs. generalist pollinators: though generalists may indeed show a tendency in our data to interact with exotic plants (Memmott & Waser 2002), generalist pollinators must be more likely to interact with any given plant tautologically. Therefore, it is critically important to determine whether any observed relationship is truly relevant ecologically and above and beyond what would be expected solely by chance.

To perform the above-described comparison, we compared the empirically observed tendencies to the same tendencies for 100 networks whose interactions have been randomized. We randomized the empirical networks using the swap method that shuffles interactions between species while simultaneously preserving each species' total number of interactions (Fortuna *et al*. 2010). By randomizing the networks in this way, we also explicitly maintain each network's overall probability of connecting to an exotic plant, implying that the only noteworthy differences across randomizations should be the identities of the pollinators that interact with the exotic plants.

Lastly, to statistically quantify how the probability that a pollinator is connected to an exotic plant varied depending on the pollinator's degree or contribution to nestedness, we used a mixed-effects logistic regression (Zuur *et al*. 2009) that takes the form:

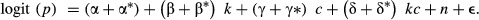
eqn 1

Here, the dependent variable *p* is the probability of interacting with an exotic plant, and the independent variables include each pollinator's ranked degree *k*, ranked nestedness contribution *c*, a statistical interaction between the two and a random effect *n* for network identity that accounts for underlying variation between networks in the overall tendency for pollinators to interact with exotic plants. The coefficient α defines the model intercept, ε is the model residual, β quantifies the effect of species' degree, γ the effect of nestedness contribution and δ the strength of the interaction effect. Lastly, the coefficients α*, β*, γ* and δ* quantify the differences between the empirical and randomized data.

## Results

### Identification of exotic plant species

Within the set of 2230 flowering plants present across all networks in our data set (of which 1746 were taxonomically unique), we conclusively identified a total of 48 exotic plant species (of which 29 were taxonomically unique) (Table1). These plants were present within 25 of the 59 networks; geographically, these 25 networks also came from six of the seven major continents (Fig.1). Thirteen of the 25 networks featured a single exotic species, seven included two exotic plants, one included three exotic plants, three included four exotic plants, and the remaining network had six exotic plants (Table S1).

**Table 1:** Exotic plant species identified within our data set of 59 empirical pollination networks. For each species, we also indicate the location where it was classified as exotic and the number of networks in that location in which it was observed

Exotic species	Location where exotic	Number of networks
*Aegopodium podagraria*	Denmark	2
*Ageratum conyzoides*	Galapagos Islands (Ecuador)	1
*Bidens pilosa*	Galapagos Islands (Ecuador)	1
*Bidens pilosa*	Japan	1
*Calystegia sepium*	Denmark	2
*Campanula rotundifolia*	Denmark	2
*Cirsium arvense*	New Zealand	1
*Cirsium arvense*	United Kingdom	2
*Cytisus scoparius*	New Zealand	1
*Daucus carota*	Denmark	2
*Daucus carota*	United Kingdom	1
*Eupatorium cannabinum*	Denmark	2
*Hieracium pilosella*	New Zealand	1
*Leucaena leucocephala*	Mauritius	1
*Leucanthemum vulgare*	Azores (Portugal)	1
*Linaria vulgaris*	United States	1
*Lotus corniculatus*	Azores (Portugal)	1
*Opuntia stricta*	Spain	1
*Oxalis corniculata*	Japan	2
*Oxalis pes-caprae*	Canary Islands (Spain)	1
*Oxalis pes-caprae*	Greece	1
*Passiflora foetida*	Galapagos Islands (Ecuador)	2
*Prunus serotina*	Denmark	1
*Psidium guajava*	Galapagos Islands (Ecuador)	1
*Scaevola frutescens*	Japan	1
*Scaevola sericea*	Mauritius	1
*Senecio lautus*	Australia	1
*Solidago sempervirens*	Azores (Portugal)	1
*Tanacetum vulgare*	Denmark	2
*Trifolium pratense*	Denmark	1
*Trifolium repens*	Denmark	3
*Trifolium repens*	Japan	3
*Trifolium repens*	New Zealand	1
*Verbascum thapsus*	New Zealand	1
*Vicia sativa*	United Kingdom	1

### Community, network and species-level differences

**Figure 2:**
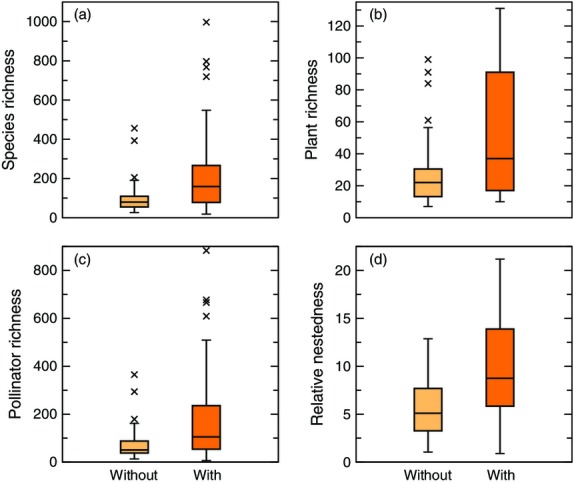
Properties of pollination networks with and without exotic plants. (a) Species richness, (b) plant richness, (c) pollinator richness and (d) relative nestedness were significantly different between the two types of communities. In all panels, the box covers the 25th–75th percentiles, the middle line marks the median, and the maximum length of the whiskers is 1.5 times the interquartile range. Points outside this range show up as outliers.

Overall, our analyses gave scattered indications that communities and networks with exotic plants exhibited characteristic differences (Fig.2). Specifically, we found significant community-level differences in terms of the total species richness (χ^2^ = 7.00, *P* = 0.008), plant richness (χ^2^ = 5.44, *P* = 0.020) and pollinator richness (χ^2^ = 6.48, *P* = 0.011); in each of these three instances, communities with exotic plants exhibited tended to exhibit greater richness across the board. In contrast, we observed no significant differences for the ratio of pollinator to plant diversity (χ^2^ = 0.90, *P* = 0.612) nor phylogenetic diversity of the plant community (χ^2^ = 1.22, *P* = 0.269).

Despite any hypothesis to the contrary (Levine *et al*. 2003), there was no indication within the data that the networks with more exotic plants also had greater endemic plant richness (*P* = 0.188). Furthermore, the relative proportion of exotic to native plants in a community was a decreasing function of total species richness (*P* = 0.018), plant richness (*P* = 0.003) and pollinator richness (*P* = 0.028). Overall, these community-level results indicate that more speciose communities are generally more likely to contain exotic plants whereas less speciose communities tend to support a greater proportion of exotic plants.

At the network level, we found no significant differences in terms of network connectance (χ^2^ = 2.77, *P* = 0.096), the average number of interactions per plant (χ^2^ = 1.70, *P* = 0.192), the average number of interactions per pollinator (χ^2^ = 1.62, *P* = 0.203) or nestedness (χ^2^ = 1.62, *P* = 0.203). On the other hand, we did observe that communities with exotic plants had significantly different relative nestedness (χ^2^ = 7.97, *P* = 0.005) in that they tended to exhibit higher values of relative nestedness.

In slight contrast to what we observed at the community and network levels, we found no evidence that exotic plants themselves exhibited characteristic differences when compared to other plants in their community. Specifically, our analyses indicated that exotic plants were statistically indistinguishable from native plants in terms of their ranks of phylogenetic uniqueness (χ^2^ = 2.34, *P* = 0.126), degree (χ^2^ = 0.154, *P* = 0.694) and nestedness contribution (χ^2^ = 1.52, *P* = 0.218). Moreover, these conclusions did not change when testing for differences between the absolute species-specific values as opposed to within-network ranks (*P* = 0.761, *P* = 0.332 and *P* = 0.404, respectively).

### Interaction-level differences

**Figure 3:**
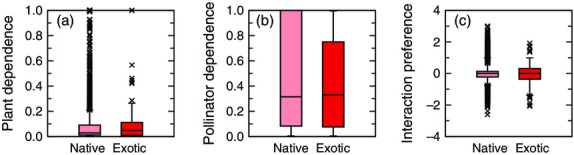
Properties of interactions between pollinators and native or exotic plants. (a) Exotic plants showed a significant tendency to have lower dependences on all of their observed pollinators than do native plants. (b) Pollinators showed a significant tendency to have higher dependence on exotic plants than on their native counterparts. (c) There were no significant differences observed between interaction preference when comparing interactions between pollinators and native plants to those between pollinators and exotic plants. All boxes are as in Fig.2.

In contrast to what we observed at the community, network and species levels, our subsequent analysis indicated statistically significant differences at the interaction level (Fig.3). When examining species' dependences, we found that pollinator species had significantly higher dependences on exotic plants than on native plants (*z*_5360_ = 20.21, *P* < 10^−4^). For exotic plants, we observed the opposite trend, such that exotic plants had, on average, significantly lower dependences than their native counterparts (*z*_5360_ = −6.60, *P* < 10^−4^). These differences in species' dependences would appear to indicate that interactions between pollinators and exotic plants are fundamentally different. However, when extending our test of this hypothesis by examining interaction preferences, we found no additional statistical support; that is, interactions between pollinators and exotic plants showed no tendency to occur more or less often than would be expected by random chance (*t*_5360_ = 1.31, *P* = 0.187).

### Pollinator-level differences

**Figure 4:**
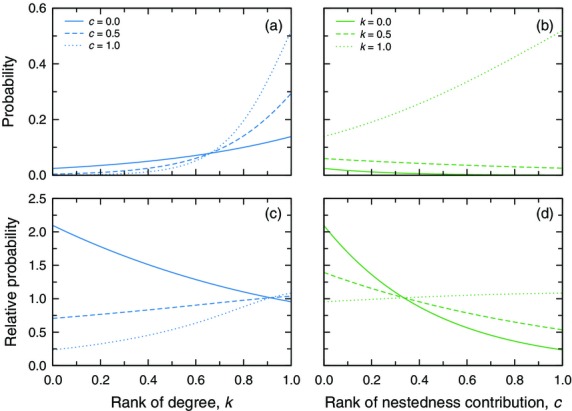
Identifying the pollinator species that are most likely to interact with exotic plants. (a, b) As a pollinator's degree or nestedness contribution increases, so does the probability that it interacts with an exotic plant. The strength of the observed tendency for degree increases significantly as you move from a pollinator with the lowest nestedness contribution (*c* = 0; solid line), to one with the median contribution (*c* = 0.5; dashed line), and to the strongest contributor (*c* = 1.0; dotted line). Similarly, the observed tendency for nestedness contribution increases significantly as you move from the most specialist pollinator (*k* = 0.0; solid line), to a moderate specialist/generalist pollinator (*k* = 0.5; dashed line) to the most generalist pollinator (*k* = 1.0; dotted line). (c, d) When compared to the same tendencies in randomized networks, the relative probability (i.e., the ratio between the predicted probability for the empirical data and the predicted probability for the randomized data) is greatest for the most specialist pollinators or those with the lowest nestedness contribution. All else being equal, specialist pollinators that are weak contributors to nestedness interact with exotic plants in the empirical networks far more often than would be expected at random. Because of the complex structure of our mixed-effects logistic regression model, we only plot the model-based predictions in all panels.

Our mixed-effects logistic regression of the empirical networks gave indications of multiple significant predictors of the probability that a pollinator interacts with an exotic plant (Table2 and Fig.4). Both a pollinator's degree and nestedness contribution appear to play a significant role, and there is also a significant, positive interaction between these two pollinator attributes.

**Table 2:** Results of mixed-effects logistic regression model to predict the probability that a pollinator interacts with an exotic plant. For each model predictor, we show the parameter estimate, its *z*-value and the associated *P*-value. To facilitate comparison between the predicted responses for the empirical and randomized networks, we also specify the network ‘type’ to which each parameter estimate corresponds

Network type	Predictor	Parameter	Estimate	*z*-value	*P*-value
Empirical	Intercept	α	−3.69	−13.91	< 10^−4^
Empirical	Degree	β	1.86	8.14	< 10^−4^
Empirical	Nestedness contribution	γ	−3.69	−18.69	< 10^−4^
Empirical	Degree × Nestedness contribution	δ	5.60	21.96	< 10^−4^
Random	Intercept	α+α^*^	−4.44	−19.11	< 10^−4^
Random	Degree	β+β^*^	2.67	11.70	0.0004
Random	Nestedness contribution	γ+γ^*^	−1.49	−7.40	< 10^−4^
Random	Degree × Nestedness contribution	δ+δ^*^	3.17	12.05	< 10^−4^

Focusing first on pollinator degree, we find that the larger it is the more likely it is that the pollinator interacts with an exotic plant. This result itself may be rather unsurprising as it is akin to a confirmation of what it means to be a generalist. The significant interaction, however, implies that the ‘generalist behaviour’ of generalist pollinators becomes stronger as we move from the smallest nestedness contributor in a community to the largest (and despite the fact that degree and nestedness contribution are only weakly correlated). Moving to pollinator nestedness contribution, we find that there is a significant negative relationship between contribution and the probability that the most specialist pollinators interact with exotic plants. However, this relationship becomes significant and positive for more generalist pollinators such that, the larger the pollinator's nestedness contribution, the higher the probability it interacts with an exotic plant. Generalizing across all of the empirical networks, the most likely pollinator to interact with an exotic plant is one that is both highly generalist *and* a strong contributor to nestedness.

Notably, we also observed comparable relationships between the probability of interacting with an exotic plant and a pollinator's degree and its nestedness contribution across the ensemble of randomized networks (Table2). Similar to what we observed in the empirical data, the relationship with degree was significant and positive, and the strength of this relationship increased with increasing nestedness contribution of the pollinator. Likewise, the relationship with nestedness contribution in the randomized networks shifted from significantly negative for poorly connected pollinators to significantly positive for the most connected pollinators; overall, however, these patterns for nestedness contribution were generally weaker in the randomized networks than in the empirical networks.

Since we observed significant relationships in both the empirical and randomized networks, we also directly compared the two to gain additional insight regarding which pollinators in a community are most likely to interact with an exotic plant (Fig.4). Intriguingly, this comparison indicated that the pattern in the empirical webs was significantly different from that in the randomized webs. In particular, weak contributors to nestedness in the empirical networks are more likely to interact with exotic species than similar species in the randomized networks. On the other hand, strong contributors to nestedness in the empirical networks are less likely to interact with exotic species than comparable species in the randomized networks. In addition, there is a significant interaction between degree and nestedness contribution such that poorly connected pollinators that are also weak contributors to nestedness are significantly more likely to interact with exotic plants in the empirical networks than would be expected by random chance alone.

## Discussion

Across our globally distributed data set of pollination networks, we found multiple threads of evidence to support the suggestion that community properties provided a significant predictor of communities with non-native plant species (Rejmánek & Richardson 1996; Naeem *et al*. 2000; Kennedy *et al*. 2002; Fridley 2010). Somewhat paradoxically, we found that both plant richness and pollinator richness were positively associated with the presence of exotic plants, despite the fact that the two are thought to influence plant–plant competition in opposite ways (Levine 2000; Levine *et al*. 2003; Mitchell *et al*. 2009; Powell, Chase & Knight 2011). On the other hand, we found no indication that the exotic plants themselves were different than their native counterparts (e.g. in terms of their generalism or phylogenetic uniqueness).

Previous research on pollination networks has also indicated a strong link between network structure and species' coexistence (Bascompte, Jordano & Olesen 2006; Thébault & Fontaine 2010; Saavedra *et al*. 2011; Saavedra & Stouffer 2013). In this regard, we found a single result here that suggests that network-driven hypotheses could indeed provide a ‘mechanism’ which is exploited by exotic plants: networks with exotic species present exhibit significantly larger relative nestedness. This result could potentially help explain the aforementioned effects of plant and pollinator richness since it has been shown analytically that nestedness maximizes indirect facilitation between flowering plants (or pollinators) relative to competition (Bastolla *et al*. 2009) as well as tends to maximize individual species' abundances (Suweis *et al*. 2013).

Here, we also identified clear differences with regard to how exotic plants' interactions were distributed across the pollinator community. On one hand, exotic plants exhibited significantly lower dependences on their pollinators than did native species; conversely, pollinators showed significantly higher dependences on exotic plants. The underlying asymmetry of this relationship is broadly consistent with earlier research that concluded that mutualistic relationships tend to be asymmetric in nature (Bascompte, Jordano & Olesen 2006; Aizen, Morales & Morales 2008), whether the result of coevolution within mutualistic communities (Thompson 1994) or because of variation in species' relative abundances (Vázquez *et al*. 2007). The exotic nature of the flowering plants studied here, however, would appear to rule out the former hypothesis while future tests of the latter would require additional abundance data that are based on independent observations as opposed to the interaction matrix (Staniczenko, Kopp & Allesina 2013; García *et al*. 2014).

Above all else, the most consistent pattern we observed was in the network attributes of pollinator species that interact with exotic plants. When compared to the random expectation, the data indicated in particular that there was a significant tendency for exotic plants to interact with specialist pollinators that are also weak contributors to nestedness. This pattern is particularly intriguing since theoretical research has suggested that specialists should be the most vulnerable to extinction (Allesina 2012) but that being a weak contributor to nestedness can counterbalance this effect (Saavedra *et al*. 2011). Viewed from this perspective, it is possible that the establishment of exotic plants is, in fact, *beneficial* for these otherwise highly vulnerable specialist pollinators. As future studies begin to compile better resolved data regarding the long-term dynamics of mutualistic communities (Burkle, Marlin & Knight 2013), we expect there to be more conclusive evidence regarding the positive impacts of non-native plants.

Within this present study, we have focused on the question of whether network structure facilitates or prevents establishment of exotic plant species, or whether exotic plants exhibit characteristic differences when compared to their native counterparts. In doing so, we have adopted an explicitly macroecological perspective (Trojelsgaard & Olesen 2013) while setting aside the related question of whether and how non-native plants modify the resulting network structure of the pre-existing community (Moragues & Traveset 2005; Aizen, Morales & Morales 2008; Bartomeus, Vilà & Santamaría 2008; Jakobsson, Padrón & Traveset 2009). Unfortunately, this latter question is best addressed by studying paired networks (Albrecht *et al*. 2014) across an equally large geographic extent and is therefore beyond the possibilities provided by our present data set. Methodologically, we also recognize that the testing of some of our hypotheses could have been hampered by aspects of the data set (Moran 2003); examples include the proportion of networks with exotic species to those without, an imbalance of native to exotic species, or a lack of abundance data with which to distinguish between ‘superabundant’ invasive plants and exotic plants as a whole. The breadth of interrelated patterns we have uncovered here, however, suggests multiple promising avenues for more detailed or mechanistic studies in the future.

Thinking more broadly, all plant species are constantly adopting new strategies with which to respond to competition for pollination (Moeller 2004; Mitchell *et al*. 2009). Along these lines, it has previously been shown that natural selection ‘within a network context’ (Fontaine 2013) should favour a reduction of effort towards interactions that negatively influence plants’ short- and long-term fitness (Thompson 1994; Zhang, Hui & Terblanche 2011; Suweis *et al*. 2013). Consequently, sufficiently strong competition should hypothetically lead to coevolutionary selection and adaptation *away* from interactions with pollinators who themselves are most vulnerable to extinction (Slobodkin 1964) – specialists (Allesina 2012) and strong contributors to nestedness (Saavedra *et al*. 2011). It is therefore an enticing possibility that the patterns we have observed for exotic plants appear because these species fill otherwise vacant ‘coevolutionary niches’ that are the natural result of the internal dynamic of mutualistic networks.

## Data accessibility

All data studied here are publicly available via the Web of Life Database (http://www.web-of-life.es), GISD – the Global Invasive Species Database (http://www.issg.org/database/welcome/) and GISIN – the Global Invasive Species Information Network (http://www.gisin.org).

## Supporting Information

**Table S1.** Empirical data sets analysed.
