# Supplementary material for: How exotic plants integrate into pollination networks
Source: J Ecol. 2014 Sep 18;102(6):1442–50. doi: 10.1111/1365-2745.12310 (PMC4277853; doi:10.1111/1365-2745.12310)
Supplement: Supplementary file 1 — Table S1. Empirical data sets analysed. [file jec0102-1442-sd1.pdf]

Table S1 Empirical datasets analyzed. We show the network, location of study, pollinator richness, plant richness, number of exotic plants identified, and the network's original reference. All pollination networks were obtained via the Web of Life database (http://www.web-of-life.es).

Network	Location	Pollinators	Plants	Exotic plants	Reference
1	Chile	101	84	0	Arroyo *et al.* (1982)
2	Chile	64	43	0	Arroyo *et al.* (1982)
3	Chile	25	36	0	Arroyo *et al.* (1982)
4	Canada	102	12	0	Barrett & Helenurm (1987)
5	United States	275	96	1	Clements & Long (1923)
6	United Kingdom	61	17	2	Dicks *et al.* (2002)
7	United Kingdom	36	16	1	Dicks *et al.* (2002)
8	Canary Islands (Spain)	38	11	0	Dupont *et al.* (2003)
9	Sweden	118	24	0	Elberling & Olesen (1999)
10	Greenland	76	31	0	Elberling & Olesen (unpubl.)
11	Mauritius	13	14	2	Olesen *et al.* (2002)
12	Canary Islands (Spain)	55	29	0	Olesen (unpubl.)
13	South Africa	56	9	0	Ollerton *et al.* (2003)
14	Canada	81	29	0	Hocking (1968)
15	Greece	666	131	1	Petanidou (1991)
16	Spain	179	26	0	Herrera (1988)
17	United Kingdom	79	25	1	Memmott (1999)
18	Denmark	105	39	6	Olesen (unpubl.)
19	Australia	85	40	1	Inouye & Pyke (1988)
20	Canada	91	20	0	Kevan (1970)
21	Japan	677	91	1	Kato *et al.* (1990)
22	Argentina	45	21	0	Medan *et al.* (2002)
23	Argentina	72	23	0	Medan *et al.* (2002)
24	Canada	18	11	0	Mosquin & Martin (1967)
25	United States	44	13	0	Motten (1982, 1986)
26	Galapagos Islands (Ecuador)	54	105	4	McMullen (1993)
27	New Zealand	60	18	0	Primack (1983)
28	New Zealand	139	41	4	Primack (1983)
29	New Zealand	118	49	1	Primack (1983)
30	Venezuela	53	28	0	Ramirez & Brito (1992)
31	Venezuela	49	48	0	Ramirez (1989)
32	United States	33	7	0	Schemske *et al.* (1978)
33	Canada	34	13	0	Small (1976)
34	Chile	128	26	0	Smith-Ramirez *et al.* (2005)
35	Jamaica	36	61	0	Percival (1974)
36	Azores (Portugal)	12	10	3	Olesen (unpubl.)
37	Denmark	40	10	2	Montero (2005)
38	Denmark	42	8	0	Montero (2005)
39	Canary Islands (Spain)	51	17	1	Stald (2003)
40	Jamaica	43	29	0	Ingversen (2006)
41	Dominica	43	31	0	Ingversen (2006)
42	Galapagos Islands (Ecuador)	6	12	1	Philipp *et al.* (2006)
43	Denmark	82	28	4	Montero (2005)

(Continued on next page)

(Continued from previous page)

Network	Location	Pollinators	Plants	Exotic plants	Reference
44	Japan	609	110	2	Kato (2000)
45	Greenland	26	17	0	Lundgren & Olesen (2005)
46	Denmark	44	16	0	Bundgaard (2003)
47	Denmark	186	19	1	Dupont & Olesen (2009)
48	Denmark	236	30	2	Dupont & Olesen (2009)
49	Denmark	225	37	2	Bek (2006)
50	Canary Islands (Spain)	35	14	0	Stald (2003)
51	Argentina	90	14	0	Vázquez (2002)
52	Greenland	39	15	0	Witt (1998)
53	Japan	294	99	0	Yamazaki & Kato (2003)
54	Japan	318	113	2	Kakutani *et al.* (1990)
55	Japan	195	64	1	Kato & Miura (1996)
56	Japan	365	91	0	Kato (1993)
57	Japan	883	114	1	Inoue *et al.* (1990)
58	Spain	81	32	1	Bartomeus *et al.* (2008)
59	Brazil	13	13	0	Bezerra *et al.* (2009)

References

Arroyo, M. T. K., Primack, R. B. & Armesto, J. J. (1982). Community studies in pollination ecology in the high temperate Andes of central Chile. I. Pollination mechanisms and altitudinal variation. *Amer. J. Bot.*, **69**, 82–97.

Barrett, S. C. H. & Helenurm, K. (1987). The reproductive biology of boreal forest herbs. I. Breeding systems and pollination. *Can. J. Bot.*, **65**, 2036–2046.

Bartomeus, I., Vilà, M. & Santamaría, L. (2008). Contrasting effects of invasive plants in plant–pollinator networks. *Oecologia*, **155**, 761–770.

Bek, S. (2006). *A pollination network from a Danish forest meadow*. Master's thesis, Univeristy of Aarhus, Denmark.

Bezerra, E. L., Machado, I. C. & Mello, M. A. R. (2009). Pollination networks of oil-flowers: a tiny world within the smallest of all worlds. *J. Anim. Ecol.*, **78**, 1096–1101.

Bundgaard, M. (2003). *Tidslig og rumlig variation i et plante-bestøvernetværk.*. Master's thesis, Univeristy of Aarhus, Denmark.

Clements, F. E. & Long, F. L. (1923). *Experimental pollination; an outline of the ecology of flowers and insects*. Carnegie Institution of Washington, Washington, D.C.

Dicks, L. V., Corbet, S. A. & Pywell, R. F. (2002). Compartmentalization in plant-insect flower visitor webs. *J. Anim. Ecol.*, **71**, 32–43.

Dupont, Y. L., Hansen, D. M. & Olesen, J. M. (2003). Structure of a plant-flower-visitor network in the high-altitude sub-alpine desert of Tenerife, Canary Islands. *Ecography*, **26**, 301–310.

Dupont, Y. L. & Olesen, J. M. (2009). Ecological modules and roles of species in heathland plant-insect flower visitor networks. *J. Anim. Ecol.*, **78**, 346–353.

Elberling, H. & Olesen, J. M. (1999). The structure of a high latitude plant-flower visitor system: the dominance of flies. *Ecography*, **22**, 314–323.

Elberling, H. & Olesen, J. M. (unpubl.).

Herrera, J. (1988). Pollination relationships in southern Spanish Mediterranean shrublands. *J. Ecol.*, **76**, 274–287.

Hocking, B. (1968). Insect-flower associations in the high Arctic with special reference to nectar. *Oikos*, **19**, 359–387.

Ingversen, T. T. (2006). *Plant-pollinator interactions on Jamaica and Dominica: The centrality, asymmetry and modularity of networks*. Master's thesis, Univeristy of Aarhus, Denmark.

Inoue, T., Kato, M., Kakutani, T., Suka, T. & Itino, T. (1990). Insect-flower relationship in the temperate deciduous forest of Kibune, Kyoto: an overview of the flowering phenology and the seasonal pattern of insect visits. *Contr. Biol. Lab. Kyoto Univ.*, **27**, 377–463.

Inouye, D. W. & Pyke, G. H. (1988). Pollination biology in the Snowy Mountains of Australia: Comparisons with montane Colorado, USA. *Aust. J. Ecol.*, **13**, 191–205.

Kakutani, T., Inoue, T., Kato, M. & Ichihashi, H. (1990). Insect-flower relationship in the campus of Kyoto University, Kyoto: an overview of the flowering phenology and the seasonal pattern of insect visits. *Contr. Biol. Lab. Kyoto Univ.*, **27**, 465–521.

Kato, M. (1993). Flowering phenology and anthophilous insect community in the cool-temperate subalpine forests and meadows at Mt. Kushigata in the central part of Japan. *Contr. Biol. Lab. Kyoto Univ.*, **28**, 119–172.

Kato, M. (2000). Anthophilous insect community and plant-pollinator interactions on Amami Islands in the Ryukyu Archipelago, Japan. *Contr. Biol. Lab. Kyoto Univ.*, **29**, 157–252.

Kato, M., Kakutani, T., Inoue, T. & Itino, T. (1990). Insect-flower relationship in the primary beech forest of Ashu, Kyoto: an overview of the flowering phenology and the seasonal pattern of insect visits. *Contr. Biol. Lab. Kyoto Univ.*, **27**, 309–375.

Kato, M. & Miura, R. (1996). Flowering phenology and anthophilous insect community at a threatened natural lowland marsh at Nakaikemi in Tsuruga, Japan. *Contr. Biol. Lab. Kyoto Univ.*, **29**, 1–48.

Kevan, P. G. (1970). *High Arctic insect-flower relationships: The interrelationships of arthropods and flowers at Lake Hazen, Ellesmere Island, Northwest Territories, Canada.*. Ph.D. thesis, University of Alberta, Edmonton, Canada.

Lundgren, R. & Olesen, J. M. (2005). The dense and highly connected world of Greenland's plants and their pollinators. *Arct. Antarct. Alp. Res.*, **37**, 514–520.

McMullen, C. K. (1993). Flower-visiting insects of the Galapagos Islands. *Pan-Pac. Entomol.*, **69**, 95–106.

Medan, D., Montaldo, N. H., Devoto, M., Mantese, A., Vasellati, V., Roitman, G. G. & Bartoloni, N. H. (2002). Plant-pollinator relationships at two altitudes in the Andes of Mendoza, Argentina. *Arct. Antarct. Alp. Res.*, **34**, 233–241.

Memmott, J. (1999). The structure of a plant-pollinator food web. *Ecol. Lett.*, **2**, 276–280.

Montero, A. C. (2005). *The ecology of three pollination networks*. Master's thesis, Univeristy of Aarhus, Denmark.

Mosquin, T. & Martin, J. (1967). Observations on the pollination biology of plants on Melville Island, N.W.T., Canada. *Can. Field Nat.*, **81**, 201–205.

Motten, A. F. (1982). *Pollination ecology of the spring wildflower community in the deciduous forest of Piedmont North Carolina.*. Ph.D. thesis, Duke University, USA.

Motten, A. F. (1986). Pollination Ecology of the Spring Wildflower Community of a Temperate Deciduous Forest. *Ecol. Monogr.*, **56**, 21–42.

Olesen, J. M. (unpubl.).

Olesen, J. M., Eskildsen, L. I. & Venkatasamy, S. (2002). Invasion of pollination networks on oceanic islands: importance of invader complexes and endemic super generalists. *Divers. Distrib.*, **8**, 181–192.

Ollerton, J., Johnson, S. D., Cranmer, L. & Kellie, S. (2003). The pollination ecology of an assemblage of grassland asclepiads in South Africa. *Ann. Bot.*, **92**, 807–834.

Percival, M. (1974). Floral ecology of coastal scrub in southeast Jamaica. *Biotropica*, **6**, 104–129.

Petanidou, T. (1991). *Pollination ecology in a phryganic ecosystem*. Ph.D. thesis, Aristotelian University, Thessaloniki, Greece.

Philipp, M., Bcher, J., R. Siegismund, H. & R. Nielsen, L. (2006). Structure of a plant-pollinator network on a pahoehoe lava desert of the Galpagos Islands. *Ecography*, **29**, 531–540.

Primack, R. B. (1983). Insect pollination in the New Zealand mountain flora. *New Zeal. J. Bot.*, **21**, 317–333.

Ramirez, N. (1989). Biología de polinización en una comunidad arbustiva tropical de la Alta Guayana Venezolana. *Biotropica*, **21**, 319–330.

Ramirez, N. & Brito, Y. (1992). Pollination biology in a palm swamp community in the Venezuelan Central Plains. *Bot. J. Lin. Soc.*, **110**, 277–302.

Schemske, D. W., Willson, M. F., Melampy, M. N., Miller, L. J., Verner, L., Schemske, K. M. & Best, L. B. (1978). Flowering ecology of some spring woodland herbs. *Ecology*, **59**, 351–366.

Small, E. (1976). Insect pollinators of the Mer Bleue peat bog of Ottawa. *Can. Field Nat.*, **90**, 22–28.

Smith-Ramirez, C., Martínez, P., Nuñez, M., González, C. & Armesto, J. J. (2005). Diversity, flower visitation frequency, and generalism of pollinators in temperate rain forests of Chiloé Island, Chile. *Bot. J. Linn. Soc.*, **147**, 399–416.

Stald, L. (2003). *Struktur og dynamik i rum og tid af et bestøvningsnetværk på Tenerife, De Kanariske Øer*. Master's thesis, Univeristy of Aarhus, Denmark.

Vázquez, D. P. (2002). *Interactions among introduced ungulates, plants, and pollinators: A field study in the temperate forest of the southern Andes*. Ph.D. thesis, University of Tennessee, Knoxville.

Witt, P. (1998). BSc thesis, University of Aarhus, Denmark.

Yamazaki, T. & Kato, M. (2003). Flowering phenology and anthophilous insect community in a grassland ecosystem at Mt. Yufu, western Japan. *Contr. Biol. Lab. Kyoto Univ.*, **29**, 255–318.
